# Unfolding the Spatial and Temporal Neural Processing of Making Dishonest Choices

**DOI:** 10.1371/journal.pone.0153660

**Published:** 2016-04-20

**Authors:** Delin Sun, Tatia M. C. Lee, Zhaoxin Wang, Chetwyn C. H. Chan

**Affiliations:** 1 Laboratory of Neuropsychology, The University of Hong Kong, Hong Kong, China; 2 Laboratory of Cognitive Affective Neuroscience, The University of Hong Kong, Hong Kong, China; 3 The State Key Laboratory of Brain and Cognitive Sciences, The University of Hong Kong, Hong Kong, China; 4 Key Laboratory of Brain Functional Genomics (MOE & STCSM), Institute of Cognitive Neuroscience, School of Psychology and Cognitive Science, East China Normal University, Shanghai, China; 5 Applied Cognitive Neuroscience Laboratory, Department of Rehabilitation Sciences, The Hong Kong Polytechnic University, Hong Kong, China; Beijing Normal University, CHINA

## Abstract

To understand the neural processing that underpins dishonest behavior in an economic exchange game task, this study employed both functional magnetic resonance imaging (fMRI) and event-related potential (ERP) methodologies to examine the neural conditions of 25 participants while they were making either dishonest or honest choices. It was discovered that dishonest choices, contrary to honest choices, elicited stronger fMRI activations in bilateral striatum and anterior insula. It also induced fluctuations in ERP amplitudes within two time windows, which are 270–30 milliseconds before and 110–290 milliseconds after the response, respectively. Importantly, when making either dishonest or honest choices, human and computer counterparts were associated with distinct fMRI activations in the left insula and different ERP amplitudes at medial and right central sites from 80 milliseconds before to 250 milliseconds after the response. These results support the hypothesis that there would be distinct neural processing during making dishonest decisions, especially when the subject considers the interests of the counterpart. Furthermore, the fMRI and ERP findings, together with ERP source reconstruction, clearly delineate the temporal sequence of the neural processes of a dishonest decision: the striatum is activated before response, then the left insula is involved around the time of response, and finally the thalamus is activated after response.

## Introduction

Dishonest behavior is a common social phenomenon in everyday living [1, 2] and as such there has been an increasing momentum in research on understanding the neural underpinnings of dishonest behavior [3–7]. In a socioeconomic situation, dishonest behavior can be presented as attempts to increase one’s profits at the expense of other people’s well-being through intentionally utilizing the victims’ ignorance of the truth [8]. Previous studies on social decision making, e.g., reciprocity, trust, fairness, have shown that various processes of specific neural processing are involved when an individual begins to consider the interests of his or her counterparts (for a review, see Rilling and Sanfey [9]). Thus, it is both interesting and important to understand whether the neural processing involved during the making of dishonest decisions is different when a decision maker does or does not consider the interests of the counterpart. This knowledge may enhance our current understanding of the inner neural mechanisms related to dishonesty in real life. Unfortunately, very few studies looked into that comparison.

In this study, we investigated the neural correlates of dishonest decisions through employing an economic exchanging game task. This task paradigm was adapted from the classical Trust Game [10] and is able to imitate dishonest behaviors and the thought processes underpinning them in real life, such as those of occupying another person’s property, taking advantage of the victim’s ignorance, and running the risk of being caught and punished for dishonesty. The dishonest choices could consist of two processes, including the “participant’s self-serving intention” (i.e., whether to occupy the counterpart’s benefits) and “the risk of the action itself” (i.e., whether the choice will be detected), and we did not aim to differentiate them in this study. Therefore, a “dishonest choice” denoted a choice with a self-serving intention that was risky and led to either large reward (i.e., not detected) or no reward (i.e., detected and punished). In contrast, an “honest choice” meant a choice without a self-serving intention that always resulted in medium reward. Our previous behavioral studies have shown that this task could soundly replicate the process of choosing to make dishonest decisions [11]. In the task, the participant was asked to interact with his or her human and computer counterparts. Most importantly, the participant was told that a dishonest choice will lead to the reduction in the income of the human counterpart. In contrast, dishonest and honest choices would not affect the computer counterpart. Furthermore, participants in our study were not asked to attempt manipulating the truth because we were interested in the neural processing of making a dishonest decision. Hence, we avoided confusion caused by fabrication of information.

In our task paradigm, as in many social interactions, a dishonest choice is associated with either larger gains or larger losses compared to the consequences of an honest choice. Therefore, anticipation of a dishonest (versus honest) choice may elicit either positive or negative feelings. Previous studies have shown that the insula is activated by either positive or negative feelings [12]. Furthermore, a decision to commit to dishonest behavior when interacting with human beings is often associated with reducing others’ profits to increase self-benefits and is therefore antisocial (helpful dishonesty and white lies are not discussed here [4, 13]). This is because playing dishonestly against human contradicts mutual cooperation in society [9, 14], which is crucial for the functioning of the society. Guilt may thus affect the dishonest individual, which is not ideal [15, 16]. Moreover, humans are, by nature, empathetic with regard to the experiences of other individuals [17, 18]. Thus, an individual who plays dishonestly against human may foresee the potential losses suffered by the victim and, hence, suffer from regret and other negative emotions. Researchers have found that stronger activations in the insula were triggered by conditions that violate social norms [19–21] (however, see van den Bos et al. [22]) and by empathizing with others’ pains [23]. Furthermore, previous studies have shown that increased insula activations often precede safe choices [24] and the rejection of buying high-priced products [25]. All of these evidences have supported the role of insula in responding to either positive or negative feelings and in making decisions. We thus hypothesized to find different insula activations between making dishonest and honest choices, and also hypothesized that the dishonest- and honest-related insula activations are modulated by the counterpart type.

Our second aim was to investigate when the neural processing during dishonest decisions occurs, in addition to where the neural processing occurs. Previous studies on dishonest decisions had often employed neuroimaging methodologies such as fMRI (functional magnetic resonance imaging). Their high spatial resolution has bettered our understanding of the neural substrates when a dishonest choice is made. On the other hand, their low temporal resolution (e.g., about 2–3 seconds for fMRI) has prevented us from investigating the dynamic changes of the neural responses, especially when a dishonest decision is made very quickly [26, 27]. In this study, all participants were invited to take part in both fMRI and ERP (event-related potential) recordings (in different sessions). The two methods contributed to the determination of neural correlates, using both high spatial resolution (a few millimeters, or mm) and high temporal resolution (about a millisecond, or ms). In one of our previous studies, the combination of these two methods successfully delineated a neural model of lying on face familiarity [28].

Neurophysiological study on dishonest decisions has been scarce, and little is known about the quick neural processing (about hundreds of ms) preceding the making of a dishonest choice. Previous studies on deception through using memorized words [29], different attitudes [30] and face familiarity [28] have shown that ERPs within 300 ms before responses were different when deceptive and non-deceptive responses were made. Therefore, we also hypothesized that different ERP amplitudes could be detected before dishonest and honest choices were made within the interval 300 ms.

Our third aim was to delineate a neural model to describe the dynamic interactions between temporal and spatial neural processing in the dishonest decisions. The fMRI and ERP data were acquired via the test conducted on the same group of volunteers employing the same task paradigms. Thus, the participants might have utilized similar cognitive/affective strategies across sessions. We hypothesized that, within the before-response intervals determined by ERP data, the brain regions determined by fMRI data would show different source intensities for dishonest and honest choices.

## Materials and Methods

This research has been approved by The Ethics Committee in the East China Normal University, Shang Hai, China.

### Participants

Twenty-six Chinese female university students in the East China Normal University, China, participated in both fMRI and ERP sessions. Only females were recruited to avoid the potential gender difference in decision making [11, 31]. All of the participants were right-handed [32] with normal or corrected-to-normal vision. None of them reported a history of physical, neurological, or mental disorders. All participants gave written informed consent for a protocol approved by the local ethics committee. One participant was excluded due to technical problems during ERP data recording. Finally, 25 participants (20~25 years old) were included in data analyses.

### Experimental design

Each participant received instructions before the experiment: “You will play in an online game as one of the trustees interacting with anonymous investors. Half of the investors will be human beings and the other half will be computer programs. A photo of either a human face or a laptop will be presented during each trial to indicate the type of the investor (human or computer) without other information. You should treat each trial as a single-shot interaction since players won’t recognize each other. In each trial, you will receive monetary investment from an investor, then repay a proportion of the increased investment and hold the rest. You will also receive a proposal from the investor on how to divide the reward. You should choose by yourself to repay as much as (honest choice) or less than (dishonest choice) the portion proposed. After that, a computer center will decide whether or not with 5:5 chance to show the real information to the investor. You will win nothing in the trial if a dishonest choice is caught but will gain your portion in the other conditions. The expected utility (reward × possibility) will be indifferent between a dishonest choice and an honest choice. As a result, a human investor will obtain less than or equal to the portion that he or she should get according to the proposal, depending on whether the choice is a dishonest or an honest one. On the contrary, there will be no actual payment to a computer investor. All human players finally will receive real monetary bonuses proportional to the amounts earned during the experimental sessions.” After receiving the abovementioned information, the participants did not know that, in fact, they were actually always playing a computer counterpart and never playing a human counterpart.

**Fig 1:**
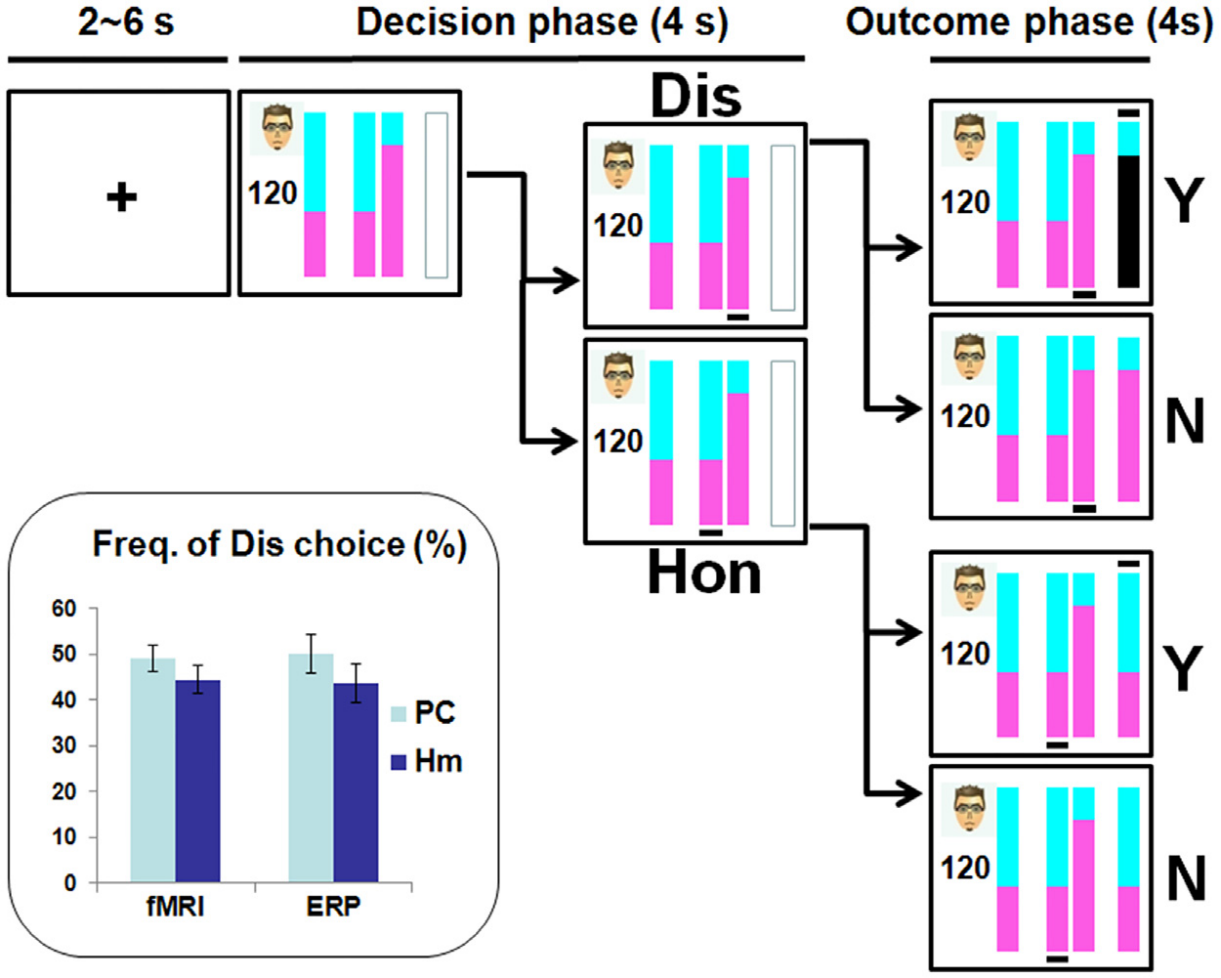
Task paradigm and behavioral performances. In each trial, after an inter-trial-interval (2~6 s), a dishonest or an honest choice was made within the Decision phase and was followed by a feedback in the Outcome phase. The portions of rewards assigned to the counterpart (investor) and the participant (trustee) were respectively represented by the cyan and purple areas in the vertical stacked bars. The frequencies of dishonest choices were shown in the lower left corner. Participants made fewer dishonest choices when interacting with human than computer investors. Dis = dishonest choice, Hon = honest choice, Hm = human investor, PC = computer investor, Y = detected, N = not detected. Error bar denotes the standard error of the mean.

In this task, the portions of reward assigned to the counterpart and the participant were respectively represented by the cyan and purple areas in the vertical stacked bars shown in the screen. In each trial (for a schemata of the task, see Fig 1), after a fixation presented for 2~6 s, the amount of the increased investment (i.e., the total amount to be divided) and the proposal bar were shown on the left-hand side for 4 s (i.e., Decision phase) during which the participant had to make her choice by choosing (through pressing one of two buttons with the right index or middle finger) between the two bars in the middle of the screen. One of the two bars was consistent with the proposal by the counterpart (i.e., honest bar), whereas the other bar (i.e., dishonest bar) indicated a plan advantageous to the participant herself. The two bars were matched in positions by the two response buttons. During the Decision phase, once the response was made, a black line appeared under the chosen bar. If there was no response, or should the response exceed the Decision phase, all reward would be sent to the counterpart. In the following 4 s (i.e., Outcome phase), the participant was informed of whether the real situation was detected by the counterpart and how much she gained.

During the Outcome phase, a black line appeared above the outcome bar if the real situation was detected; while no line was shown if the detection did not occur. The probability of detection was always 50%. When a dishonest choice was detected, the participant gained nothing in this trial and her area in the outcome bar became black. Under the other conditions, the participant kept the portion of herself. The human investors’ faces consisted of 2 males and 2 females (of frontal view with neutral expression), and were shown randomly. The permutation of the total amount, i.e., the amount to be divided (80~150), the proposed portion of repayment to the counterpart (60%, 65%, 70%), and the location (left or right) of the dishonest and honest bars were randomized within each participant. These values were adapted from those in our previous study [11] and were clearly justified in the supplementary document: S1 Supporting Information.

### Experimental procedure

Each participant was asked to participate in two sessions (within 2 weeks) in which Blood-oxygen-level dependent (BOLD) signals or scalp electrical signals were collected. The order of the two sessions was counterbalanced across participants. Each participant practiced 8~15 trials before the formal experiment to familiarize herself with the task paradigm. Moreover, to reduce the ERP artifacts caused by eye movement, she was trained to blink only within the inter-trial-interval. In the formal task, there were 5 runs of 24 trials in the fMRI session and 12 runs of 24 trials in the ERP session. More trials were included in the ERP session to compensate for the relatively low signal-to-noise ratio in ERP data recording. After completing the two sessions, each participant was asked to fill in a questionnaire including the question ‘How many human counterparts do you feel that you have played against in this game?’ They were debriefed and received 250 Chinese yuan (no difference across participants because of the local ethical requirements) as compensation, including the bonus for the task. She was not allowed to know the other participants’ income.

### Data acquisition

Neuroimaging scans were conducted on a 3-Tesla Siemens Trio MR scanner. Thirty-five axial slices covering the whole brain were obtained using a T2*-weighted echo planar imaging (EPI) sequence (TR = 2000 ms, TE = 30 ms, flip angle = 90°, matrix = 64×64, Field of View (FOV) = 240×240 mm^2^, slice thickness = 4 mm without gap) for functional images. The axial slices were adjusted to be parallel to the AC-PC plane. A high-resolution structural image for each participant was also acquired using 3D MRI sequences (TR = 1900 ms, TE = 3.43 ms, flip angle = 7°, matrix = 256×256, FOV = 210×210 mm^2^, slice thickness = 1 mm). The visual stimuli presentations and response collections were performed through the integrated functional imaging system (IFIS).

Scalp electrical potentials were recorded through an elastic electroencephalogram (EEG) cap (Brain Products Company, Germany) embedded with 64 tin scalp electrodes according to the extended international 10–20 system. All channel recordings were referenced to a channel at the vertex, and all channel impedances were kept below 10 kΩ. The EEG signals were amplified using a 0.05–100 Hz band-pass filter and continuously sampled at 500 Hz. Vertical and horizontal eye movements were recorded by two electrodes at the temporal and lower sides of the left eye, respectively. The visual stimuli presentations and response collections were performed through E-Prime software (Psychology Software Tools, Inc.).

### Data analysis

#### Behavioral data

Considering that participants made choices in more than 95% of the trials in both sessions, the trials without responses or response exceeding the Decision phase were dropped from analyses. The reaction time in both sessions was log-transformed to correct for its skewed distribution, and was analyzed with a repeated measures analysis of variance (ANOVA) model with three within-subjects factors, i.e., Session (fMRI vs. ERP), Choice (dishonest vs. honest) and Investor (human vs. computer). The frequency of dishonest choice (= 1 –frequency of honest choice) in both sessions was entered into a repeated measures ANOVA model with two within-subjects factors, i.e., Session (fMRI vs. ERP) and Investor (human vs. computer). Results were considered statistically significant at *p* < 0.05. Post hoc analysis with Bonferroni correction was employed for significant interactions. Behavioral data were analyzed using SPSS version 20.0 software (IBM Corp.).

#### fMRI data

The SPM8 software (Wellcome Department of Imaging Neuroscience, UK) was employed for the preprocessing of both the neuroimaging and neurophysiological data, as well as for the fMRI, ERP, and source reconstruction analyses. The functional scans of each participant were spatially realigned to adjust for head movement and corrected for slice-acquisition timing. Anatomical images were then co-registered to the mean functional image and were segmented into grey/white matter according to an anatomical template of Eastern Asian brains. After that, the functional images were normalized to the Montreal Neurological Institute (MNI) brain template and smoothed with an 8-mm full-width half-maximum (FWHM) Gaussian filter.

In this study, we were only interested in the processing of decision making. The findings (both fMRI and ERP) during the outcome presentation are reported elsewhere [33]. The general line model (GLM) was used to examine the experimental effects across task events. Four regressors were employed to model the 4s-duration of the Decision phase. They were combinations of Choice (dishonest vs. honest) and Investor (human vs. computer). A regressor modeling the onset of button pressing and six extra regressors modeling residual head motions were also included. These regressors were convolved with the SPM canonical hemodynamic response function. High-pass temporal filtering with a cut-off of 128 s was recruited to remove low-frequency drifts. The parameter estimates (βs) for each condition per run were calculated for all brain voxels. They were then used to form the contrasting images of the four experimental conditions, which were the combinations of Choice (dishonest vs. honest) and Investor (human vs. computer).

The contrasting images from all participants were then group-level analyzed through a two-way ANOVA model with 2 within-subject factors, i.e., Choice and Investor. Results of whole brain analyses survived peak-level FWE (family wise error) correction (*p* < 0.05) within the whole brain. Results of the interaction effect between Choice and Investor was voxel-level height thresholded at *p* < 0.001 with cluster size of ≥ 20 voxels, and survived cluster-level FWE correction (*p* < 0.05) within bilateral insula. The WFU Pickatlas toolbox (http://www.nitrc.org/projects/wfu_pickatlas/) was used to generate a mask image of bilateral insula. The xjView toolbox (http://www.alivelearn.net/xjview) was used for the anatomical definition. The MarsBaR toolbox (http://marsbar.sourceforge.net/) was used to extract the % signal change for each task condition from each participant, if necessary. The % signal changes were analyzed using a repeated measures analysis of variance (ANOVA) model with two within-subjects factors, i.e., Choice (dishonest vs. honest) and Investor (human vs. computer).

#### ERP data

The continuous recordings were filtered (0.1–30 Hz), corrected for eye-movement [34], cut into two types of epochs (-200~300 ms post stimulus onset, and -3800~500 ms post button press), and corrected for baseline signals (-200~0 ms post stimulus onset and -3800~-3600 ms post response corresponding to the above two types of epochs, respectively). The two types of epochs were utilized to investigate the neural responses that were time-locked to either stimulus onset or response. In order to remove the effects of motor response from the dataset time-locked to stimulus onset, the length of stimulus-locked epochs (except for its baseline) was shorter than the shortest reaction time across trials and across participants. Furthermore, to make sure that the baselines of the epochs time-locked to response were not influenced by the early neural processing during the Decision phase, the length of the response-locked epoch (except for its baseline) was longer than the longest reaction time across trials and across participants. Epochs containing amplitudes exceeding ±100 μV were removed. Epochs were averaged for each task condition and for each participant through using the robust averaging method [35]. An additional low-pass filter (< 30 Hz) was employed to remove the high-frequency noises elicited by the robust averaging. The ERP data were then re-referenced to a computed average of the whole-scalp EEG channels. After these steps, the ERP datasets were down-sampled to 100 Hz and were converted into three-dimensional images by linear interpolation. The x, y and z dimensions reflect “left-right”, “anterior-posterior” and “early-late”, respectively. The images were smoothed by an FWHM of [9 mm, 9 mm, 20 ms] [28]. The statistical analyses for ERP data were similar to those for the fMRI data. The results were voxel-level height thresholded at *p* < 0.01 and survived cluster-level FWE correction (*p* < 0.05) within the whole scalp × time space of interest (0~300 ms for stimulus-locked data, and -300~300 ms for response-locked data, respectively).

#### fMRI-informed ERP source reconstruction

The source reconstruction analysis was recruited to test whether the possible brain sources (revealed by fMRI results) were activated within the time intervals (defined by ERP results) for the contrasts of interest [28]. It is based on the group inversion (imaging) method [36]. We first matched an SPM template head model to standard scalp electrode positions of the extended 10–20 system, given that we did not record each individual’s EEG channel locations due to machine errors. We then employed the boundary element model (BEM) for the forward model calculation. Datasets related with the effects of interest, i.e., (I) conditions (human & honest + human & dishonest) and (computer & honest + computer & dishonest) for the main effect of Investor, (II) conditions (human & dishonest + computer & dishonest) and (human & honest + computer & honest) for the main effect of Choice and (III) conditions (human & dishonest + computer & honest) and (human & honest + computer & dishonest) for the interaction effect between Choice and Investor, in each participant were investigated by the Multiple Sparse Priors (MSP) approach [37] within the time windows of interest. The source locations were further restricted within spheres (radius = 24 mm) in which the center was located at the peak significance detected by fMRI data [38]. For each participant, the intensity of source activity for each task condition within the time window of interest was converted into the brightness of a 3D image which could be superimposed onto a standard MNI brain template. Finally, after spatial smoothing with a FWHM of 12 mm, images were analyzed with paired t tests. Absolute statistical values were reported because this source analysis did not reflect the polarity of contrasts. The results were voxel-level height thresholded at *p* < 0.05 (uncorrected). This lenient threshold was recruited because our aim was to test whether there was a difference of source intensities between conditions for the contrasts of interest around the spatial priors, but not to explore within the whole brain.

## Results

### Behavioral findings

The trial number for each task condition in either fMRI or ERP sessions was shown in Table 1. We found that choices in the ERP session (1166.5±57.3 ms) were made quicker (*F*(1,24) = 21.460, *p* < 0.001) than in the fMRI session (1291.7±52.8 ms). This difference might be due to the training effect caused by more trials in the ERP session than in the fMRI session. Importantly, no significant interaction effect involving the factor Session was found for the frequency of dishonest choices or for the reaction time (*F*s < 1, *p*s > 0.5), suggesting that the participants’ choices were consistent between sessions.

**Table 1:** Trial Number for each task condition in either fMRI or ERP sessions.

	PC & Hon	Hm & Hon	PC & Dis	Hm & Dis
*fMRI session*				
Mean	29.6	32.4	28.8	25.9
Std	8.2	9.3	9.2	9.1
*ERP session*				
Mean	71.2	80.0	71.7	62.8
Std	29.8	28.8	29.7	30.6

Notes: Dis = dishonest choice; Hon = honest choice; Hm = human investor; PC = computer investor.

No significant main effect of Session (*F*(1,24) = 0.005, *p* = 0.946) was found for frequency of choice. Fewer dishonest choices (*F*(1,24) = 4.533, *p* = 0.044) were made for human (44.1±3.4% averaged across sessions) than computer (49.6±3.0% averaged across sessions) investors (as shown in Fig 1, lower left corner). Moreover, a significant interaction effect between Choice and Investor was found for the reaction time (*F*(1,24) = 4.801, *p* = 0.038). However, post hoc analyses did not show significant difference between dishonest and honest choices whatever the counterpart was a computer (*F*(1,24) = 0.008, *p* = 0.929) or a human (*F*(1,24) = 2.723, *p* = 0.112). For the behavioral data collected within the ERP session, the shortest and longest reaction times across trials and across participants were 334 ms and 3516 ms, respectively. These values were used to define epochs for ERP data analyses.

Twelve participants reported that they felt that they had played against 1–5 human counterparts during the tasks. Eleven participants reported that the number of human counterparts was above five. Two participants reported that they believed that there were several human counterparts, although they could not feel their counterparts because of the indirect interaction.

### fMRI findings

**Fig 2:**
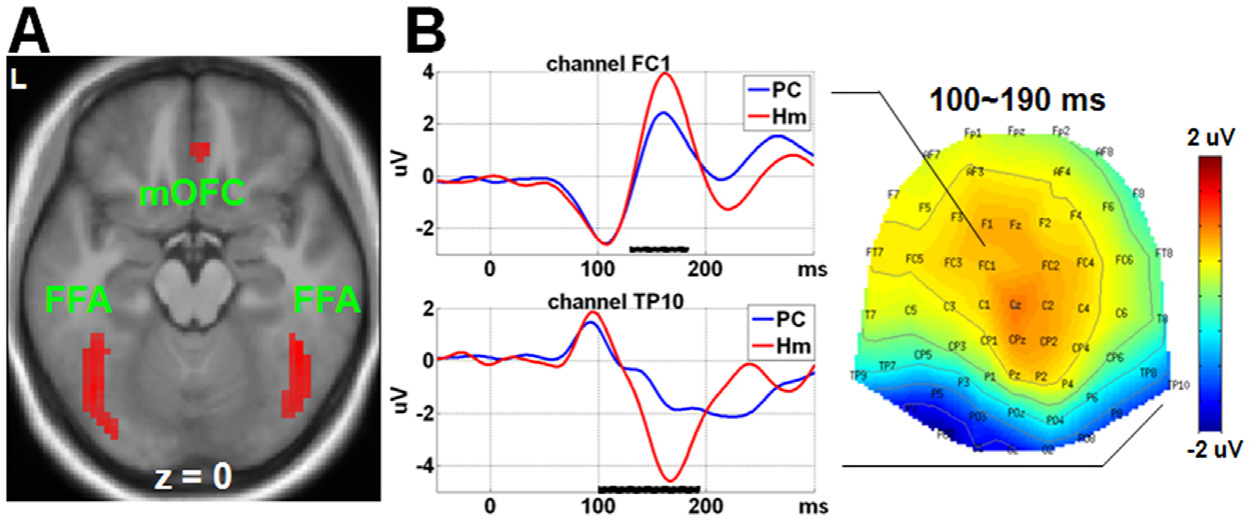
fMRI and ERP findings for the main effect of Investor. Human (Hm) versus computer (PC) investor elicited (A) stronger activations in bilateral fusiform face areas (FFA) and right medial orbitofrontal cortex (mOFC) (*p* < 0.05 FWE correction, cluster ≥ 20 voxels), and (B) more positive-going amplitudes in the frontal-central sites (close to FC1) and more negative-going amplitudes in the right occipito-temporal sites (close to TP10) within 100~190 ms time-locked to stimulus. The SPM T map (for fMRI data) and the scalp topography (for ERP data) represented the contrast of (Hm-PC). The waveforms in the representative channel are shown for the ERP findings. The horizontal black bars represent the time windows detecting statistical significance. Here, 0 ms indicates the time point of stimulus onset. L = left.

**Fig 3:**
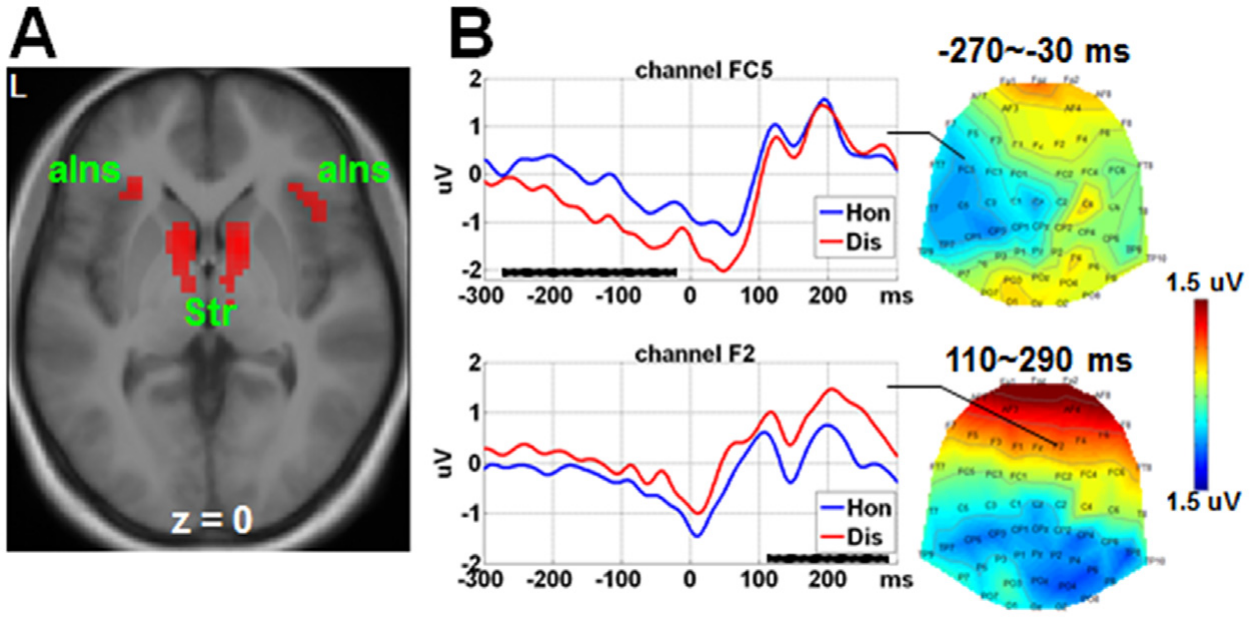
fMRI and ERP findings for the main effect of Choice. Dishonest (Dis) versus honest (Hon) choices elicited (A) stronger activations in bilateral striatum (Str) and anterior insula (aIns) (*p* < 0.05 FWE correction, cluster ≥ 20 voxels), and (B) more negative-going amplitudes within -270~-30 ms in the left frontal-central sites (close to FC5), and more positive-going amplitudes within 110~290 ms in the right frontal sites (close to F2). The SPM T map (for fMRI data) and the scalp topography (for ERP data) represented the contrast of (Dis-Hon). The waveforms in the representative channel are shown for the ERP findings. The horizontal black bars represent the time windows detecting statistical significance. Here, 0 ms indicates the time point of response. L = left.

Results of fMRI analyses were shown in Table 2. Human versus computer investors elicited stronger BOLD signals in bilateral fusiform gyrus, right medial orbitofrontal cortex, right lingual gyrus, left superior frontal gyrus, and bilateral precuneus (Fig 2A). Dishonest versus honest choices elicited stronger BOLD activations in the left thalamus, bilateral striatum, bilateral anterior insula, and right supplementary motor area (Fig 3A). No stronger activations were detected for honest than dishonest choices. No stronger activations were detected for computer than human investors.

**Table 2:** The fMRI results.

Brain Area	Cluster	Z	x	y	z
**Main effect of Choice**					
*Dis > Hon*					
L Tha	415	6.166	-3	-13	7
R Str		6.151	12	5	-2
L Str		5.922	-12	8	-3
L Ins (BA13)	23	5.330	-27	29	-2
R Ins (BA13)	70	5.329	42	23	4
R SMA (BA6)	23	5.236	9	8	58
*Dis < Hon*					
NS					
**Main effect of Investor**					
*Hm > PC*					
R FFA (BA37)	228	6.290	45	-49	-17
L FFA (BA37)	256	6.022	-42	-64	-17
R mOFC (BA11)	88	5.470	9	44	-14
R Lin (BA18)	21	5.222	6	-88	-5
L SFG (BA9)	37	5.220	-9	50	37
L/R Pre (BA31)	46	5.010	0	-55	31
*Hm < PC*					
NS					
**Interaction between Choice and Investor**					
*Hm(Dis-Hon) > PC(Dis-Hon)*					
NS					
*Hm(Dis-Hon) < PC(Dis-Hon)*					
L Ins (BA13)	20	3.770	-39	2	-5

Notes: The effects of Choice and Investor survived peak-level FWE correction (*p* < 0.05) within the whole brain. Their interaction effect survived cluster-level FWE correction (*p* < 0.05) in bilateral insula. Cluster = number of voxels within the cluster; Z = Z value; BA = Brodmann’s area; L = left; R = right; FFA = fusiform face area; Ins = insula; Lin = lingual gyrus; mOFC = medial orbitofrontal cortex; Pre = precuneus; SFG = superior frontal gyrus; SMA = supplementary motor area; Tha = thalamus; Str = striatum; Dis = dishonest choice; Hon = honest choice; Hm = human investor; PC = computer investor.

**Fig 4:**
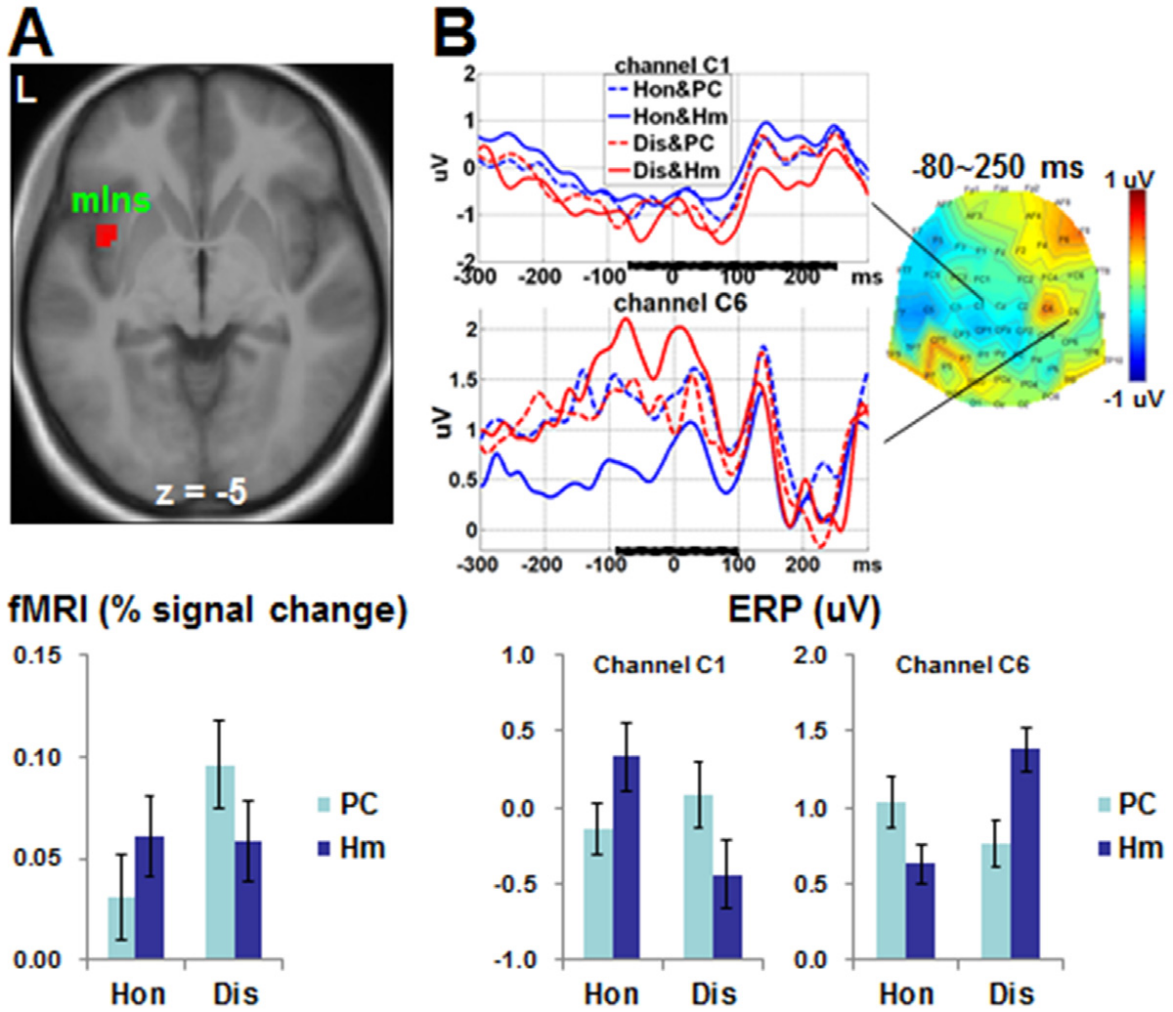
fMRI and ERP findings for interaction between Choice and Investor. (A) In left middle insula, there was a trend that human versus computer counterpart was associated with increased activations when making honest choices, and human versus computer counterpart was related with decreased activations when playing dishonestly. (B) Within the time window of -80~250 ms time-locked to response, human versus computer counterpart was associated with less negative-going amplitudes in channel C1 and less positive-going amplitudes (nonsignificant trend) in channel C6 when honest choices were made. By contrast, human versus computer counterpart was associated with more negative-going amplitudes in channel C1 and more positive-going amplitudes in channel C6 when dishonest choices were made. The SPM T map (for fMRI data) and the scalp topography (for ERP data) represented the contrast of [Hm(Dis-Hon)-PC(Dis-Hon)]. The waveforms in the representative channel are shown for the ERP findings. The horizontal black bars represent the time windows detecting statistical significance. Here, 0 ms indicates the time point of response. Error bar denotes standard error of the mean. L = left.

An interaction between Choice and Investor was found in left middle insula (Fig 4A). The % signal changes extracted from the cluster in the left middle insula showed that there was a trend that human versus computer counterpart was associated with increased activations when making honest choices (*t*(24) = 2.037, *p* = 0.053), and human versus computer counterpart was related with decreased activations when playing dishonestly (*t*(24) = -2.612, *p* = 0.015). Dishonest versus honest choices were associated with stronger activations when playing against computer counterpart (*t*(24) = 5.493, *p* < 0.001), while there was no significant difference between dishonest and honest choices when interacting with human counterparts (*t*(24) = -0.188, *p* = 0.853).

### ERP findings

Results of ERP analyses were shown in Table 3. Human versus computer investor elicited more positive-going amplitudes in the frontal-central sites (close to FC1) and more negative-going amplitudes in the right occipito-temporal sites (close to TP10) within 100~190 ms time-locked to stimulus (Fig 2B). No other effects were found significant for the stimulus-locked data.

**Table 3:** The ERP findings.

Time (ms)						
t1	tp	t2	Scalp Area	Channel	Cluster	Z
**Time-locked to stimulus onset**						
**Main effect of Choice**						
NS						
**Main effect of Investor**						
*Hm > PC*						
130	160	190	Frontal- Central	FC1	1813	5.684
*Hm < PC*						
100	160	190	R Temporal-Parietal	TP10	1039	4.868
**Interaction between Choice and Investor**						
NS						
**Time-locked to response**						
**Main effect of Choice**						
*Dis > Hon*						
110	250	290	R Frontal	F2	1468	3.394
*Dis < Hon*						
-270	-210	-30	L Frontal-Central	FC5	1642	3.481
**Main effect of Investor**						
NS						
**Interaction between Choice and Investor**						
*Hm(Dis-Hon) > PC(Dis-Hon)*						
-80	60	100	R Central	C6	1073	3.567
*Hm(Dis-Hon) < PC(Dis-Hon)*						
-70	-60	250	Central	C1	1543	3.403

Note: All results were height thresholded at *p* < 0.01 and survived cluster-level FWE correction (*p* < 0.05). t1, t2 and tp represent the beginning, ending and peaking time (ms) of the cluster, respectively; Channel = EEG channel nearest to the peak significance; Cluster = number of voxels within the cluster; Z = Z value; L = left; R = right; Dis = dishonest choice; Hon = honest choice; Hm = human investor; PC = computer investor.

For the response-locked data, no significant result was found for the effect of Investor. Dishonest versus honest choices elicited more negative-going amplitudes in the left frontal-central sites (close to channel FC5) within the time window -270~-30 ms. Moreover, in a time interval post response, i.e., 110~290 ms, dishonest versus honest choices elicited more positive-going amplitudes in the right frontal sites (close to channel F2, see Fig 3B).”

An interaction between Choice and Investor was found in the central sites (close to C1 within -70~250 ms, and close to C6 within -80~100 ms; Fig 4B). The mean amplitudes extracted from the two channels within the corresponding time windows showed that human versus computer counterpart was associated with less negative-going amplitudes in channel C1 (*t*(24) = 2.536, *p* = 0.018) and less positive-going amplitudes (nonsignificant trend) in channel C6 (*t*(24) = -1.937, *p* = 0.065) when honest choices were made. By contrast, human versus computer counterpart was associated with more negative-going amplitudes in channel C1 (*t*(24) = -2.254, *p* = 0.034) and more positive-going amplitudes in channel C6 (*t*(24) = 2.099, *p* = 0.047) when dishonest choices were made.

### Source analyses findings

**Fig 5:**
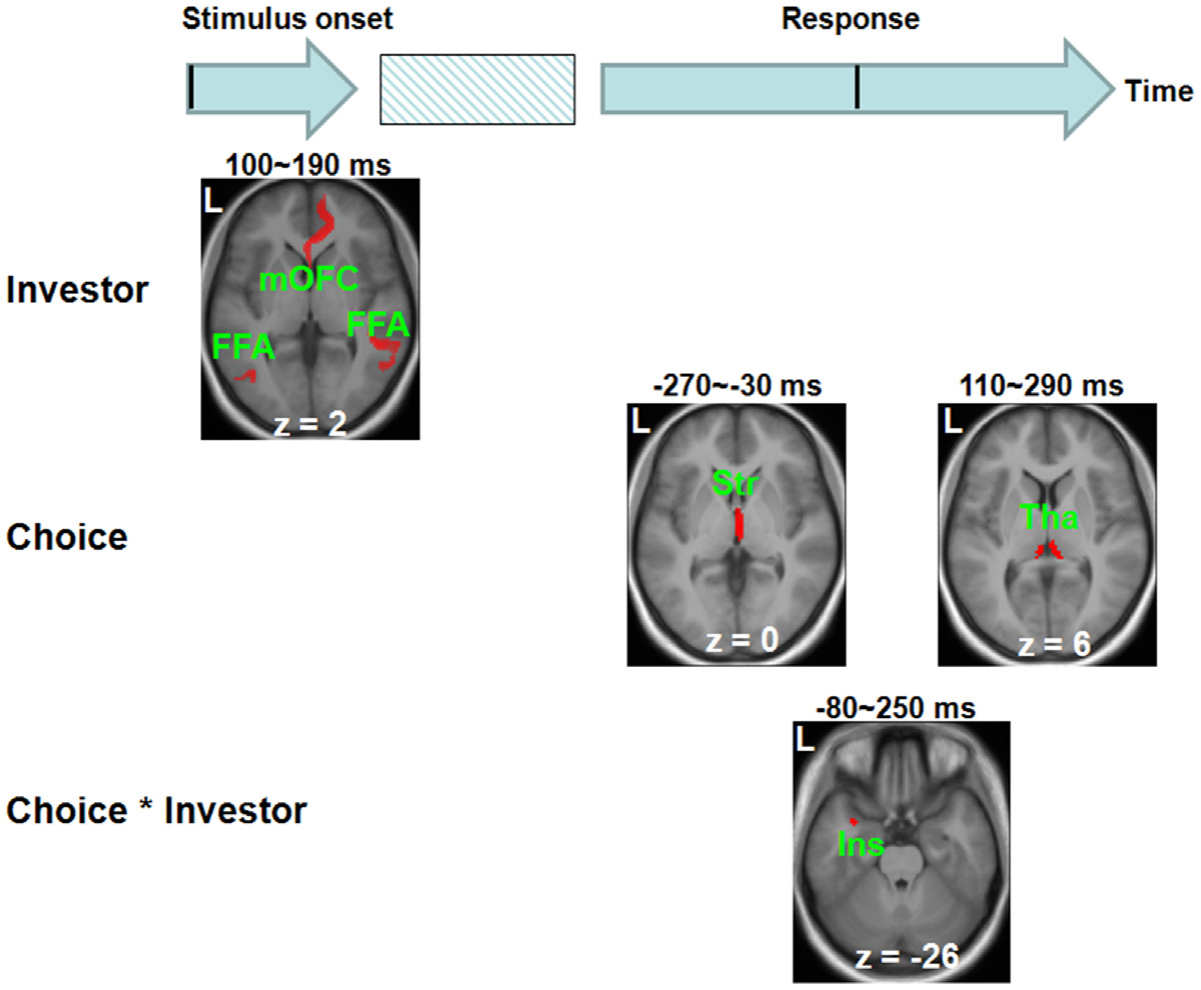
Source reconstruction analyses results. Human and computer investors elicited different source intensities in brain regions close to medial orbitofrontal cortex (mOFC) and bilateral fusiform face areas (FFA) within 100~190 ms post stimulus onset. Honest and dishonest choices elicited different source intensities in brain regions close to striatum (Str) within -270~-30 ms and in areas close to thalamus (Tha) within 110~290 ms post response. The interaction between Choice and Investor was found in brain regions close to left insula (Ins) within -80~250 ms post response. Results are height-thresholded at *p* < 0.05 with cluster size > 20 voxels. L = left.

The source analyses results were shown in Fig 5 and Table 4. Human and computer investors elicited different source intensities in brain regions close to medial orbitofrontal cortex and bilateral fusiform face areas within 100~190 ms post stimulus onset. Honest and dishonest choices elicited different source intensities in brain regions close to striatum within -270~-30 ms and in areas close to thalamus within 110~290 ms post response. The interaction between Choice and Investor was found in brain regions close to left insula within -80~250 ms post response.

**Table 4:** Source reconstruction analyses findings.

Brain Area	Cluster	Z	x	y	z
**Main effect of Investor (100~190 ms stimulus-locked)**					
L mOFC	1301	2.942	-10	32	-12
R mOFC	553	2.533	14	30	-26
R Lin (BA18)	631	2.510	30	-88	-8
L FFA (BA37)	364	2.335	-32	-80	-2
R FFA (BA37)	124	2.259	48	-54	-2
**Main effect of Choice (-270~-30 ms response-locked)**					
Str	334	2.271	2	-20	-6
**Main effect of Choice (110~290 ms response-locked)**					
L Tha	30	1.769	-8	-36	2
R Tha	49	1.717	6	-30	4
**Interaction between Choice and Investor (-80~250 ms response-locked)**					
L Ins (BA13)	26	1.933	-34	4	-26

Note: All results were height thresholded at *p* < 0.05 with cluster size > 20 voxels. The findings were shown here without considering the direction of the contrasts. The anatomical labels of the brain regions were based on the nearest fMRI spatial priors employed in the source reconstruction analyses. Cluster = number of voxels within the cluster; Z = Z value; BA = Brodmann’s area; L = left; R = right; FFA = fusiform face area; Ins = insula; Lin = lingual gyrus; mOFC = medial orbitofrontal cortex; Tha = thalamus; Str = striatum.

## Discussion

Our study is the first on the neurological implications of dishonest decisions that employs both neuroimaging and neurophysiological methodologies on the same experimental task. It is also the first study to investigate the neural correlates in relation to dishonest decisions when the counterpart’s benefits are considered or not. Our findings have advanced our understanding of the neural processing of dishonest decisions in several aspects.

First, this study has contributed to the identification of the neural substrates in specific cases of dishonesty. The characteristic of the specific dishonest decision was that the decision would affect the interests of both the dishonest individual and the victim. In this study, we directly compared participants’ behavioral and neural responses to human counterparts with those to computer counterparts. The participant was informed beforehand that her choice would affect her counterpart’s income when playing against a human counterpart. However, her decision would only affect her own interest when playing against a computer counterpart. Our fMRI data showed that dishonest choices elicited stronger activations in bilateral insula than honest choices. Further, in a cluster of voxels in the left insula, stronger activations (a trend of significance) were elicited by human than computer counterpart when making an honest choice, while stronger activations were elicited by computer than human counterpart when making a dishonest choice. This finding was consistent with our first *a priori* hypothesis that dishonest- and honest-related insula activations are modulated by the counterpart type.

Given that the insula activations may be associated with either positive or negative feelings [12], there were two speculated relationships between insula activations and choices in the present study. The first was that negative feelings activate insula and drive the decision of rejecting a choice [24, 25]: (1.1) the more negative feelings (i.e. stronger insula activations) elicited by the anticipation of larger loss of a dishonest choice prevent the selection of a dishonest choice and thus precede an honest choice; (1.2) when playing against a human (versus computer) counterpart, the more negative feelings (i.e. stronger insula activations) about a dishonest choice elicited by the anticipation of social norm violation [19–21] and negative consequences to the counterpart [23] prevent the selection of a dishonest choice and thus precede an honest choice; (1.3) when playing against a computer (versus human) counterpart, the more negative feelings (i.e. stronger insula activations) about contributing little to counterpart’s benefits prevent the selection of an honest choice and thus precede a dishonest one. By contrast, the second speculation was that positive feelings activate insula and drive the decision of making a choice: (2.1) making a dishonest (versus honest) choice was associated with more positive feelings (i.e. stronger insula activations) about the anticipation of larger reward; (2.2) when playing dishonestly, a computer (versus human) counterpart was related with more positive feelings (and stronger insula activations) caused by fewer anticipations of social norm violation and counterpart’s negative consequence; (2.3) when playing honestly, a human (versus computer) counterpart is associated with positive feelings (i.e. stronger insula activations) of increasing another’s benefits. Our fMRI data showed that dishonest (versus honest) choices elicited stronger activations in bilateral anterior insula whatever the counterpart was human or computer. Further, in a cluster in the left middle insula, human (versus computer) counterparts were associated with stronger activations when playing honestly but weaker activations when playing dishonestly. These results were consistent with (1.2) and (1.3) but not (1.1), while they were consistent with all of (2.1), (2.2) and (2.3). The findings seem to suggest that the positive feelings play an important role in making dishonest/honest choices. However, we can’t exclude the roles of negative feelings in making choices.

The interaction effect between the making of the Choice and identity of the Counterpart was only detected in the middle part of the left insula. The anterior insula has been documented to be associated with all types of subjective feelings. The middle insula supposedly integrates interoceptive signals with other neural inputs to form a combined representation of homeostatically salient features with regards to the individual’s internal and external environments [39, 40]. It is possible that the participants in our study had processed the emotional aspects of dishonest choices before the information was translated into subjective feeling. On the other hand, the activations in the middle insula were consistent with the findings of a recent meta-analysis by Duerden et al. [25], which showed that brain activations in response to the perception and experience of emotions within the middle insula were left-lateralized. This further supports the validity of our results. More importantly, the left middle insula may be used to differentiate dishonest decisions with bad intentions from making general risky decisions, which are associated with anterior insula activations and are crucial for survival [41].

Stronger fMRI activations were detected for dishonest choices compared to those of honest choices in the bilateral striatum, regardless of whether the investors were humans or computers. The striatum activations were supposed to reflect the expected reward of a choice [42]. Therefore, a dishonest choice made during our study might bring forth larger expected reward than an honest choice. More importantly, activations in these areas were not significantly different between human and computer counterparts. This suggests that these areas do not reflect the neural processing during the consideration of counterpart’s benefits.

Secondly, this study also contributed to our understanding of the quick and dynamic neural responses during the making of a dishonest choice. Consistent with our second *a priori* hypothesis, the results clearly showed that dishonest choices were found to elicit more negative-going ERP amplitudes compared to honest choices in the left frontal-central sites within the interval -270~-30 ms (0 ms denotes the time of response). In accordance to our knowledge, this study is the first to report the neurophysiological findings related to dishonest decisions. The interaction between Choice and Investor was found to fall within -80~250 ms in the medial and right central sites. This depicts that when making honest choices, human versus computer counterpart was associated with less negative-going amplitudes in channel C1 and less positive-going amplitudes (nonsignificant trend) in channel C6. By contrast, when making dishonest choices, human versus computer counterpart was associated with more negative-going amplitudes in channel C1 and more positive-going amplitudes in channel C6. Time of commencement, at the peak, and at the end of this interaction effect all occurred after the time for the main effect of Choice reported above. Furthermore, the pattern of scalp distributions was also different. These results also supported the idea that the neural processing preceding dishonest decisions is different when the decision maker considers the interests of the counterpart, as compared to cases in which she does not. It is expected that after making a decision having considered the material reward and risk, lagged and distinct neural processing would be engaged to specifically deal with the conditions associated with the benefits of the counterpart. Considering that the time window of this interaction effect would have extended to even after the time of response, it is possible that some parts of the neural processing specific to the conditions associated with the counterpart’s benefits were involved, even if it contributed little to the current decision. For example, a memory was encoded for the negative feelings related to the decision to interact dishonestly with a human investor. Such neural processing may have influenced the following choices. These hypotheses need to be tested in future studies.

We have found more positive-going amplitudes when dishonest choices are made, compared to that found for honest choices in the right frontal sites within an interval post the response, i.e., 110~290 ms. A negative-going electric amplitudes deflection called error-related negativity (ERN) was detected most prominently at frontal sites within 200 ms post erroneous button press [43, 44]. A previous study by Yu and Zhou [45] found larger ERN for risky (“to bet”) than for safe (“not to bet”) choices, suggesting that the ERN plays a role in alerting the brain to the potential negative consequences of a risky action. Compared with their results, our finding showed a reversed pattern at frontal sites when the honest and dishonest choices were regarded as “not to bet” and “to bet,” respectively. This remains an open question, which is why our post-response ERP findings are different from those by Yu and Zhou [45].

Thirdly, this study has contributed to the delineation of a neural model to describe the dynamic interactions between temporal and spatial neural processing in the dishonest decisions. The fMRI and ERP data were acquired via conducting the test on the same group of volunteers employing the same task paradigms. Furthermore, our behavioral data showed that the frequency of dishonest choices (and hence also the frequency of honest choices) was similar between sessions, suggesting that participants in both data collection sessions might have used similar cognitive/affective strategies. Therefore, the neural responses recorded by both fMRI and ERP methodologies supposedly originate from the similar neural sources. Consistent with our third *a priori* hypothesis, the source analyses showed that the difference in ERP amplitudes between the dishonest and honest choices within −270~−30 ms originated from the striatum. Later, within -80~250 ms, the interaction between Choice and Investor was found to originate from the left insula. These findings suggested that, within a short interval before response, the reward-related processing is triggered when generally making a dishonest decision. Soon after its initiation, the emotion-processing is elicited specific to the condition associated with the counterpart’s interests.

The difference in ERP amplitudes between when dishonest and honest choices are made within 110~290 ms was found to originate from the thalamus. Plenty of previous studies have highlighted the roles of thalamus in arousal regulation [46]. In line with this idea, participants in our study might spend more effort to regulate arousal when making dishonest choices when compared to the case of honest choices. This explanation also aids the understanding of the differences in ERP findings between our study and that of Yu and Zhou [45]. The difference is that larger ERN in their study was supposed to reflect increased error warnings for risky choices. However, the larger frontal positivity found in our study might reflect increased regulation of arousal for dishonest choices. However, future studies should further test this idea.

The ERP results associated with the effects of Choice were all time-locked to response. By contrast, the main effect of Investor were only associated with the stimulus-locked ERPs. That is, human versus computer investor elicited more positive-going amplitudes in the frontal-central sites and more negative-going amplitudes in the right occipito-temporal sites within 100~190 ms time-locked to stimulus. This result was consistent with the ERP components named N170 and its counterpart VPP reported in previous studies on face perception [47], and was speculated to reflect the early discrimination between human and computer counterpart. The fMRI data accompanied with the source reconstruction analyses results suggested that the ERPs were originated from the bilateral medial orbitofrontal cortex and fusiform face area, also consistent with the findings of previous neuroimaging studies on face perception [48]. Given that the mean interval between stimulus and response was longer than 1000 ms, whereas the main effect of Investor was detected within 200 ms post stimulus and the effects involving Choice was found within 300 ms prior to response, it seems that the visual information about counterpart type is processed prior to the selection of a choice. However, we cannot exclude the possibility that the Choice-associated neural processing happens earlier. The onset of the neural correlates of Choice may be not time-locked to stimulus presentation and is thus difficult to be detected through the ERP method. Future studies should further uncover the temporal information of neural processing before making a dishonest choice.

Several limitations are present in this research project. The first is that only female volunteers were recruited. The lack of males hinders the general applicability of the results. Secondly, we failed to find significant sources of intensities in some brain regions as indicated by the fMRI results for the same contrast. A possible explanation is that the ERP source analyses might have ignored some activities in the brain, because the ERP (but not the fMRI) method focuses on time- and phase-locked signals. Thirdly, a laboratory experimental task paradigm was recruited in this study to control the variables. However, test subjects participating in the experiment conducted in this environment may have behaved differently compared to dishonest persons in real life. Fourthly, in this study, the dishonest choice was always entangled with being a risky choice and the honest choice was always entangled with being a sure bet. Future studies on dishonest decisions should further dissociate the neural correlates of the self-serving intention from those of the risk of the action itself, respectively. Fifthly, a computer counterpart was employed in this study to eliminate the possibility that a participant made a decision after considering either the material or immaterial interests of the counterpart. To dissociate the effect of investor type from the effect of the presence of a payment, a future study should further investigate the task condition in which the counterpart is a human whose income is not influenced by the participant’s choice. Finally, the fMRI and ERP data from the same participant were not recorded simultaneously. The time lag might have caused variability in brain activations even though the participants were the same. Future studies on the neural processing preceding dishonest decisions should document neural responses simultaneously. They should also recruit samples from different sample groups, as well as task paradigms sensitive to investigating the other cognitive/affective functions involved in dishonesty.

In summary, the findings support the hypothesis that specific neural processing is involved when making a dishonest decision, when the interests of the counterpart are considered. Further, our results provide a model describing the dynamic interactions between temporal and spatial neural processing when making a dishonest decision. That is, dishonest and honest choices elicit different neural responses in the striatum during −270~−30 ms, which may be associated with the anticipation of a reward. Later, playing honestly (versus dishonestly) against human and computer investors elicit different responses in the left insula within −80~250 ms, which may reflect the consideration of the counterpart’s benefits. Finally, dishonest and honest choices elicit different neural responses in thalamus during 110~290 ms, which may represent the regulation of arousal.

## Supporting Information

S1 Supporting Information (DOCX)

## References

[1] Hilbig BE and Zettler I. Pillars of cooperation: Honesty-Humility, social value orientations, and economic behavior. J Res Pers. 2009; 43: 516–519.

[2] Rogers CR. Toward a Modern Approach to Values—the Valuing Process in the Mature Person. J Abnorm Soc Psych. 1964; 68: 160–167. 10.1037/h0046419 14117966

[3] Vrij A. Guidelines to catch a liar In The Detection of Deception in Forensic Contexts. New York: Cambridge University Press; 2004.

[4] Abe N, Fujii T, Ito A, Ueno A, Koseki Y, Hashimoto R, et al The neural basis of dishonest decisions that serve to harm or help the target. Brain Cogn. 2014; 90C: 41–49. 10.1016/j.bandc.2014.06.005 24983819

[5] Baumgartner T, Fischbacher U, Feierabend A, Lutz K and Fehr E. The neural circuitry of a broken promise. Neuron. 2009; 64: 756–770. 10.1016/j.neuron.2009.11.017 20005830

[6] Greene JD and Paxton JM. Patterns of neural activity associated with honest and dishonest moral decisions. Proc Natl Acad Sci U S A. 2009; 106: 12506–12511. 10.1073/pnas.0900152106 19622733 PMC2718383

[7] Sip KE, Lynge M, Wallentin M, McGregor WB, Frith CD and Roepstorff A. The production and detection of deception in an interactive game. Neuropsychologia. 2010; 48: 3619–3626. 10.1016/j.neuropsychologia.2010.08.013 20727906

[8] Saxe L. Lying—Thoughts of an Applied Social-Psychologist. Am Psychol. 1991; 46: 409–415.

[9] Rilling JK and Sanfey AG. The Neuroscience of Social Decision-Making. Annu Rev Psychol. 2011; 62: 23–48. 10.1146/annurev.psych.121208.131647 20822437

[10] King-Casas B, Tomlin D, Anen C, Camerer CF, Quartz SR and Montague PR. Getting to know you: reputation and trust in a two-person economic exchange. Science. 2005; 308: 78–83. 15802598 10.1126/science.1108062

[11] Zhang HJ, Sun D and Lee TM. Impaired social decision making in patients with major depressive disorder. Brain Behav. 2012; 2: 415–423. 10.1002/brb3.62 22950045 PMC3432964

[12] Duerden EG, Arsalidou M, Lee M and Taylor MJ. Lateralization of affective processing in the insula. Neuroimage. 2013; 78: 159–175. 10.1016/j.neuroimage.2013.04.014 23587690

[13] Wu DC, Loke IC, Xu F and Lee K. Neural correlates of evaluations of lying and truth-telling in different social contexts. Brain Res. 2011; 1389: 115–124. 10.1016/j.brainres.2011.02.084 21382353 PMC3104301

[14] Fehr E and Camerer CF. Social neuroeconomics: the neural circuitry of social preferences. Trends Cogn Sci. 2007; 11: 419–427. 17913566 10.1016/j.tics.2007.09.002

[15] Koenigs M, Young L, Adolphs R, Tranel D, Cushman F, Hauser M, et al Damage to the prefrontal cortex increases utilitarian moral judgements. Nature. 2007; 446: 908–911. 17377536 10.1038/nature05631 PMC2244801

[16] Krajbich I, Adolphs R, Tranel D, Denburg NL and Camerer CF. Economic Games Quantify Diminished Sense of Guilt in Patients with Damage to the Prefrontal Cortex. J Neurosci. 2009; 29: 2188–2192. 10.1523/JNEUROSCI.5086-08.2009 19228971 PMC2646169

[17] Bernhardt BC and Singer T. The neural basis of empathy. Annu Rev Neurosci. 2012; 35: 1–23. 10.1146/annurev-neuro-062111-150536 22715878

[18] Singer T and Lamm C. The social neuroscience of empathy. Ann N Y Acad Sci. 2009; 1156: 81–96. 10.1111/j.1749-6632.2009.04418.x 19338504

[19] Montague PR and Lohrenz T. To detect and correct: Norm violations and their enforcement. Neuron. 2007; 56: 14–18. 17920011 10.1016/j.neuron.2007.09.020

[20] Rilling JK, Goldsmith DR, Glenn AL, Jairam MR, Elfenbein HA, Dagenais JE, et al The neural correlates of the affective response to unreciprocated cooperation. Neuropsychologia. 2008; 46: 1256–1266. 10.1016/j.neuropsychologia.2007.11.033 18206189

[21] Sanfey AG, Rilling JK, Aronson JA, Nystrom LE and Cohen JD. The neural basis of economic decision-making in the Ultimatum Game. Science. 2003; 300: 1755–1758. 12805551 10.1126/science.1082976

[22] van den Bos W, van Dijk E, Westenberg M, Rombouts SA and Crone EA. What motivates repayment? Neural correlates of reciprocity in the Trust Game. Soc Cogn Affect Neurosci. 2009; 4: 294–304. 10.1093/scan/nsp009 19304843 PMC2728629

[23] de Vignemont F and Singer T. The empathic brain: how, when and why? Trends Cogn Sci. 2006; 10: 435–441. 16949331 10.1016/j.tics.2006.08.008

[24] Kuhnen CM and Knutson B. The neural basis of financial risk taking. Neuron. 2005; 47: 763–770. 16129404 10.1016/j.neuron.2005.08.008

[25] Knutson B, Rick S, Wimmer GE, Prelec D and Loewenstein G. Neural predictors of purchases. Neuron. 2007; 53: 147–156. 17196537 10.1016/j.neuron.2006.11.010 PMC1876732

[26] Bechara A, Damasio H and Damasio AR. Emotion, decision making and the orbitofrontal cortex. Cereb Cortex. 2000; 10: 295–307. 10731224 10.1093/cercor/10.3.295

[27] Bechara A, Damasio H, Tranel D and Damasio AR. Deciding advantageously before knowing the advantageous strategy. Science. 1997; 275: 1293–1295. 9036851 10.1126/science.275.5304.1293

[28] Sun D, Lee TM and Chan CC. Unfolding the Spatial and Temporal Neural Processing of Lying about Face Familiarity. Cereb Cortex. 2015; 25: 927–936. 10.1093/cercor/bht284 24186897 PMC4379998

[29] Johnson R Jr., Barnhardt J and Zhu J. Differential effects of practice on the executive processes used for truthful and deceptive responses: an event-related brain potential study. Brain Res Cogn Brain Res. 2005; 24: 386–404. 16099352 10.1016/j.cogbrainres.2005.02.011

[30] Johnson R Jr., Henkell H, Simon E and Zhu J. The self in conflict: the role of executive processes during truthful and deceptive responses about attitudes. Neuroimage. 2008; 39: 469–482. 17919934 10.1016/j.neuroimage.2007.08.032

[31] Lee TM, Chan CC, Leung AW, Fox PT and Gao JH. Sex-related differences in neural activity during risk taking: an fMRI study. Cereb Cortex. 2009; 19: 1303–1312. 10.1093/cercor/bhn172 18842666 PMC2677650

[32] Oldfield RC. The assessment and analysis of handedness: the Edinburgh inventory. Neuropsychologia. 1971; 9: 97–113. 5146491 10.1016/0028-3932(71)90067-4

[33] Sun D, Chan CC, Hu Y, Wang Z and Lee TM. Neural correlates of outcome processing post dishonest choice: An fMRI and ERP study. Neuropsychologia. 2015; 68C: 148–157. 10.1016/j.neuropsychologia.2015.01.013 25582407

[34] Berg P and Scherg M. A multiple source approach to the correction of eye artifacts. Electroencephalogr Clin Neurophysiol. 1994; 90: 229–241. 7511504 10.1016/0013-4694(94)90094-9

[35] Wager TD, Keller MC, Lacey SC and Jonides J. Increased sensitivity in neuroimaging analyses using robust regression. Neuroimage. 2005; 26: 99–113. 15862210 10.1016/j.neuroimage.2005.01.011

[36] Litvak V and Friston K. Electromagnetic source reconstruction for group studies. Neuroimage. 2008; 42: 1490–1498. 10.1016/j.neuroimage.2008.06.022 18639641 PMC2581487

[37] Friston K, Harrison L, Daunizeau J, Kiebel S, Phillips C, Trujillo-Barreto N, et al Multiple sparse priors for the M/EEG inverse problem. Neuroimage. 2008; 39: 1104–1120. 17997111 10.1016/j.neuroimage.2007.09.048

[38] Henson RN, Flandin G, Friston KJ and Mattout J. A parametric empirical Bayesian framework for fMRI-constrained MEG/EEG source reconstruction. Hum Brain Mapp. 2010; 31: 1512–1531. 10.1002/hbm.20956 20091791 PMC2941720

[39] Craig AD. How do you feel—now? The anterior insula and human awareness. Nat Rev Neurosci. 2009; 10: 59–70. 10.1038/nrn2555 19096369

[40] Craig AD. Significance of the insula for the evolution of human awareness of feelings from the body. Ann N Y Acad Sci. 2011; 1225: 72–82. 10.1111/j.1749-6632.2011.05990.x 21534994

[41] Platt ML and Huettel SA. Risky business: the neuroeconomics of decision making under uncertainty. Nat Neurosci. 2008; 11: 398–403. 10.1038/nn2062 18368046 PMC3065064

[42] Knutson B and Bossaerts P. Neural antecedents of financial decisions. J Neurosci. 2007; 27: 8174–8177. 17670962 10.1523/JNEUROSCI.1564-07.2007 PMC6673081

[43] Gehring WJ, Goss B, Coles MGH, Meyer DE and Donchin E. A Neural System for Error-Detection and Compensation. Psychol Sci. 1993; 4: 385–390.

[44] Holroyd CB and Coles MG. The neural basis of human error processing: reinforcement learning, dopamine, and the error-related negativity. Psychol Rev. 2002; 109: 679–709. 12374324 10.1037/0033-295X.109.4.679

[45] Yu RJ and Zhou XL. To Bet or Not to Bet? The Error Negativity or Error-related Negativity Associated with Risk-taking Choices. J Cogn Neurosci. 2009; 21: 684–696. 10.1162/jocn.2009.21034 18564046

[46] Schiff ND. Central thalamic contributions to arousal regulation and neurological disorders of consciousness. Ann N Y Acad Sci. 2008; 1129: 105–118. 10.1196/annals.1417.029 18591473

[47] Bentin S, McCarthy G, Perez E, Puce A and Allison T. Electrophysiological studies of face perception in humans. J Cogn Neurosci. 1996; 8: 551–565. 20740065 10.1162/jocn.1996.8.6.551 PMC2927138

[48] Haxby JV, Hoffman EA and Gobbini MI. Human neural systems for face recognition and social communication. Biol Psychiatry. 2002; 51: 59–67. 11801231 10.1016/s0006-3223(01)01330-0

