# Supplementary material for: Unfolding the Spatial and Temporal Neural Processing of Making Dishonest Choices
Source: PLoS One. 2016 Apr 20;11(4):e0153660. doi: 10.1371/journal.pone.0153660 (PMC4838301; doi:10.1371/journal.pone.0153660)
Supplement: S1 Supporting Information — (DOCX) [file pone.0153660.s001.docx]

**S1 Supporting Information**

**Unfolding the Spatial and Temporal Neural Processing of Making Dishonest Choices**

Delin Sun^1,2¶^, Tatia M.C. Lee^1,2,3¶*^, Zhaoxin Wang^4^, Chetwyn C.H. Chan^5*^

^1^ Laboratory of Neuropsychology, The University of Hong Kong, Hong Kong, China.

^2^ Laboratory of Cognitive Affective Neuroscience, The University of Hong Kong, Hong Kong, China.

^3^ The State Key Laboratory of Brain and Cognitive Sciences, The University of Hong Kong, Hong Kong, China.

^4^ Key Laboratory of Brain Functional Genomics (MOE & STCSM), Institute of Cognitive Neuroscience, School of Psychology and Cognitive Science, East China Normal University, Shanghai, China.

^5^ Applied Cognitive Neuroscience Laboratory, Department of Rehabilitation Sciences, The Hong Kong Polytechnic University, Hong Kong, China.

^*^ Corresponding authors

Email: [tmclee@hku.hk](mailto:tmclee@hku.hk) (TMCL) and [chetwyn.chan@polyu.edu.hk](mailto:chetwyn.chan@polyu.edu.hk) (CCHC)

^¶^ Both authors contributed equally to this work

**Justifications of the values in the task paradigm**

The values used in the task paradigm have a solid foundation: they were from one of our published study (Zhang, Sun, and Lee, 2012), and were further tailored to fit the requirement of the present study.

1. There are four reasons for the present portions of repayment (60%, 65%, and 70%):

(1.1) the values were selected to favor both honest and dishonest behaviors, given that the participant might be lack of motivation to play dishonestly if the portion of repayment was too advantageous to her, and that the participant might be too dishonest if the portion of repayment was too disadvantageous to her.

(1.2) to keep the expected utilities the same between dishonest and honest choices, the possibility of detection was always 50%. Therefore, the reward magnitude of the dishonest choice was always twice that of the honest choice. Our values of the portions of repayment make the reward magnitudes of both honest (30%, 35% and 40%) and dishonest (60%, 70% and 80%) choices within a rational range. That is to say, there is no irrational condition in which, for example, the reward magnitude of honest choice is 60% and that of dishonest choice is beyond 100%.

(1.3) the values helped to limit the effect of inequality. In a previous fMRI study on fairness (Sanfey et al. 2003), no significant difference was found between 5:5 and 7:3 offers. In our study, the proposed portions of repayment, i.e., 60%, 65%, or 70%, were within the interval between 5:5 and 7:3 offers. Therefore, the effect caused by inequity should be very limited.

(1.4) Different values were utilized to elicit the feeling that the human counterpart was different across trials.

2. There are two reasons for the present total amount to be divided (80-150):

(2.1) the total amount varied within a smaller range to avoid the effect caused by greatly varied reward magnitudes. However, we acknowledge that this manipulation may limit our understanding of the neural responses to extremely large or small amount.

(2.2) the values were selected to elicit the feeling that the present trial was different from previous trials.

**Reference**

Sanfey, A. G., Rilling, J. K., Aronson, J. A., Nystrom, L. E., & Cohen, J. D. (2003). The neural basis of economic decision-making in the Ultimatum Game. Science, 300(5626), 1755–1758.

Zhang, H. J., Sun, D., & Lee, T. M. (2012). Impaired social decision making in patients with major depressive disorder. Brain and Behavior, 2(4), 415–423.
